# Applying Biotechnology and Bioengineering to Pediatric Lung Disease: Emerging Paradigms and Platforms

**DOI:** 10.3389/fped.2015.00045

**Published:** 2015-06-09

**Authors:** Kelley L. Colvin, Michael E. Yeager

**Affiliations:** ^1^Department of Pediatrics-Critical Care, University of Colorado Denver, Denver, CO, USA; ^2^Cardiovascular Pulmonary Research, University of Colorado Denver, Denver, CO, USA; ^3^Department of Bioengineering, University of Colorado Denver, Denver, CO, USA; ^4^Linda Crnic Institute for Down Syndrome, University of Colorado Denver, Denver, CO, USA

**Keywords:** pediatric, bioengineering, lung, extracellular matrix, stem/progenitor cell, scaffold

## Abstract

Pediatric lung diseases remain a costly worldwide health burden. For many children with end-stage lung disease, lung transplantation remains the only therapeutic option. Due to the limited number of lungs available for transplantation, alternatives to lung transplant are desperately needed. Recently, major improvements in tissue engineering have resulted in newer technology and methodology to develop viable bioengineered lungs. These include critical advances in lung cell biology, stem cell biology, lung extracellular matrix, microfabrication techniques, and orthotopic transplantation of bioartificial lungs. The goal of this short review is to engage the reader’s interest with regard to these emerging concepts and to stimulate their interest to learn more. We review the existing state of the art of lung tissue engineering, and point to emerging paradigms and platforms in the field. Finally, we summarize the challenges and unmet needs that remain to be overcome.

## Introduction

### The study of lung disease continues to be transformed by biotechnology and bioengineering

The lung is a highly complex organ that accomplishes a great variety of physiological tasks. For many chronic parenchymal and vascular lung diseases, lung transplantation, first performed in 1963 (1), becomes the last treatment option (2). Despite the progressive increase in the number of lung transplants, the number of lungs suitable for transplantation has decreased (2, 3). Mirroring this, lung transplantation for pediatric lung diseases, especially cystic fibrosis and idiopathic pulmonary hypertension, is a viable therapeutic option (4). Since the first pediatric lung transplant in 1987, the number of lung transplants performed in children is around 100 per year (5). This small number does not reflect a small clinical need, but rather is an indication of the complexities of lung transplant such as special surgical challenges related to size matching, availability of suitable donor lungs, medical center expertise, and immune system issues (6, 7). Realization of the dream of bioengineering lungs suitable for transplant in children would potentially overcome most, if not all, of these collective barriers. New tissue and cell-based techniques to study the lung, combined with the incorporation of bioengineering approaches, have the potential to increase our understanding of its complex biology, improve current therapies to obviate the need for transplant, and possibly increase the number of lungs available for transplant. Herein, we provide a short review of newer technologies and paradigms driving this exciting endeavor, with the goal to stimulate the reader toward further in-depth reading on the subject.

## Emerging Paradigms

In recent years, a number of research themes have emerged with regard to chronic lung disease. For example, the details of *how* the type of lung injury affects the downstream disease pathogenesis are much more concrete. The ability to replicate the aspects of lung pathology as close to the human condition as possible obviously relies on a deep understanding of the disease process. Pulmonary hypertension (8), lung cancer (9), chronic obstructive pulmonary disease (10), acute respiratory distress (11), and interstitial lung disease (12) are lung disorders that have been modeled extensively using *in vivo* systems, and some have utilized sophisticated *in vitro* systems [well-summarized in Ref. (13)]. Again, the extent to which any of these *in vitro* systems faithfully recapitulates the aspects of human lung disease (especially in children), or the pathobiology of animal models of lung disease, depends largely on the nature of the injury: infection (lipopolysaccharide), physical injury (hemodynamic), chemical injury (bleomycin), and hypersensitivity reactions (ovalbumin) have all been investigated (13).

In addition to the nature of the lung injury, the roles of lung endogenous and exogenous stem cells in disease processes are being increasingly investigated using *in vitro* lung modeling systems. In a recent review (14), Weiss summarized the proceedings from the 2011 Vermont Stem Cell Conference, and the reader is urged to consult this review for a glossary of stem cell terminology. The lung appears to be populated with endogenous stem and progenitor cells, from the trachea to the distal airways and alveoli. With regard to the lung *vasculature*, the precise identity and tissue location(s) of putative endogenous lung vascular stem cells have remained elusive. However, endothelial progenitor cells (circulating bone marrow-derived), fibrocytes, and mesenchymal stem cells have all been identified as having immunomodulatory and/or tissue remodeling properties in certain contexts in both the airways and the pulmonary vasculature (15). As the human lung is comprised of over 40 cell types (15), it is critically important to understand how the lung utilizes endogenous and non-lung stem or progenitor cells to maintain homeostasis and respond appropriately to injury. Indeed, the steady-state lung is extraordinarily quiescent with respect to cell proliferation, and much of what we appreciate about lung stem or progenitor cells derives from animal models of lung injury (16). Deriving stem and progenitor cells for the purpose of lung-directed therapies seems a much closer reality with the advent of strategies to differentiate embryonic stem cells and inducible pluripotent stem cells into functional lung cell lineages (17, 18).

There is an increasing awareness that cells themselves (“seeds”) represent only a portion of what makes up the collective identity of a tissue. Indeed, the functions of the extracellular matrix (ECM, “soil”) with respect to control of lung homeostasis and its impact on both endogenous and non-lung resident cells in terms of their positional and morphogenetic guidance cues are coming into sharper focus. For example, seeding of fibroblasts onto decellularized fibrotic lung results in differentiation into myofibroblasts compared to seeding on decellularized control lungs (19). Thus, the biochemical composition and the architecture of the ECM combine to provide cellular cues important for phenotypic designation, localization/addressing, and function (20). In 2008, a decellularized trachea re-seeded with primary autologous cells was transplanted to replace a small length of airway and did not require immunosuppression to remain patent (21). Numerous approaches to decellularize rodent, pig, and human lungs, as well as the information gleaned regarding the impact of ECM components on the performance of the resulting scaffolds have been extensively reviewed (15, 22, 23). Collectively, these data point to the overall feasibility of using decellularized lungs as scaffolds for tissue engineering. This could be especially important in children, given the range of lung sizes required to treat the pediatric transplant patient. These studies also have highlighted the importance of increasing our understanding of how decellularization procedures impact the lung in terms of biomechanics, ECM composition and architecture, as well as its ability to facilitate cell seeding (22).

## Emerging Platforms

**Figure 1:**
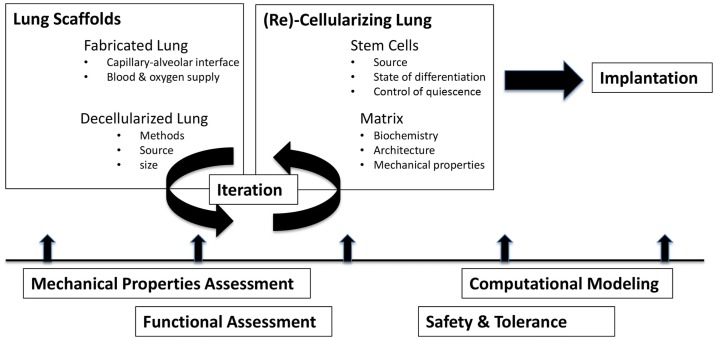
**Challenges in bioengineering lungs to treat pediatric lung disease**. Ideally, lung scaffolds of appropriate size, architecture, and complexity are prepared and then seeded with cells and/or matrix. In each stage of development, assessments of function, safety, mechanics, and efficacy each contribute to an iterative process of refinement.

Tissue engineering is a multidisciplinary process by which life sciences, materials science, and engineering principles can be married together with transplant science to achieve the goal of restoring organ and/or tissue function (24). When considering engineering any part of the lung, most approaches involve either cell therapy or utilize cell-matrix constructs (23). A smaller number of studies have focused on the development and testing of tissue-engineered scaffolds and/or cell culture devices. Regardless of approach, consideration must be given to the cell types involved (stem cells, sources of cells, etc.), the nature of the scaffolding, and the means by which the cell-matrix construct is sustained [summarized nicely in Ref. (23)]. The size of the model system (diffusion distances for gas exchange), as well as the methods used to evaluate and validate its performance, is additional critical considerations (13). Figure 1 summarizes the large variety of scientific disciplines that converge on the path toward bioengineering a lung.

### Lung scaffolds

It is generally accepted that decellularized lung scaffolds likely provide more appropriate organotypic and inductive signals by virtue of the ECM architecture (15). The methodology of decellularization (25), the amount of residual cellular or biochemical (DNA, detergent) material in the scaffold (26, 27), and the nature of the residual ECM proteins and proteoglycans (and their regional differences in the lung) (28, 29) are all important questions under intense investigation. With regard to the developing lung of a child, there may be age-specific cues in the ECM that are presently unappreciated. In addition, the use of cadaveric lungs as potential scaffolds, as well as the possibility of using animal-derived scaffolds (pig, sheep) is all avenues of active investigation. Several methods of decellularizing the lung are widely used, originating from some of the earliest efforts to decellularize rat lung (30). Regardless of technique or solution used, the goal is to remove as much cellular material and debris as possible, while maintaining as much biochemical and architectural character as possible. The use of perfusion-decellularization (31), as a means to achieve this goal, has gained prominence in recent years. Detergent-based solutions, such as sodium deoxycholate, sodium dodecyl sulfate, triton x-100, and CHAPS, can be perfused into cannulated airways and blood vessels of the lung (25). Depending on the detergents used, the duration of the procedure, and the choice of perfusion route(s), lung cells are effectively removed with minimal compromise to the vessels, airways, and lung interstitium [tabulated nicely by Wagner et al. (15)]. Due to the wide variety of approaches used, there is considerable variability in the results reported and difficulty in reproducibility and consistency of findings in the literature. Thus, direct comparison of results is problematic (32). In addition to the decellularization protocols themselves, methods of assessing the extent of decellularization also vary substantially. At present, there is no consensus regarding acceptable levels of residual cellular material, often measured by DNA content (33). Furthermore, depending on method, the ECM is altered to a greater or lesser extent during decellularization procedures as evidenced by variable collagen, elastin, and proteoglycan staining (34) and by changes in stiffness (35). Curiously, despite differences in ECM content and character, decellularized lung scaffolds all seem to accommodate reseeding with cells (36). Future studies will need to rigorously standardize decellularization techniques to move beyond methodologies based primarily on anecdotal experience.

### Fabricated devices

In addition to scaffold derived from *in vivo* sources, alternative platforms are continuously being developed. Significant challenges in design arise owing to the complexity of the lung architecture as well as the high dynamic range of functions that lung tissue accomplishes. A bioengineered lung would need to provide surface area for gas exchange over a thin non-porous but gas permeable membrane. To supply blood and oxygen to such an interface and to match ventilation with perfusion would require an even more highly complex device. Microfabrication techniques have been used to produce “organs on a chip” (37), including the lung. By layering a group of individual vascular networks with gas exchange compartments composed of polydimethylsiloxane (PDMS), a large surface area to volume ratio can be achieved (38). PDMS is an ideal material for this application given its non-porosity to water-based fluids, its permeability to gases, and its capacity to be 3-D printed and formed into microscale structures (39). Extracorporeal lung assist devices utilize such technology to provide oxygenator support for cardiac surgery and as lung assist devices for preterm infants with respiratory failure (40). In one study, a variety of blood flow rates and layering designs were tested and the resulting hydraulic resistances, oxygen transfer rates, and shear forces were calculated (38). Although scaling up to physiologically relevant sizes is accompanied by technological barriers (38), these devices have demonstrated proof of principle with regard to studying the alveolar-capillary unit (41), gas exchange (42), the effect of mechanical stretch during breathing cycles (43), and vascular networks (44, 45). Scaling up of such devices into more durable and portable designs would potentially provide continuous gas exchange support. Such biologically inspired technologies will undoubtedly continue to yield key insights into the structural and mechanical aspects of lung biology that influence interstitial fluid flow, immune-cell trafficking, and repair processes in response to injury such as edema (46). However, fabricated devices for lung oxygenation would need to fulfill several additional design criteria that are largely obviated by use of decellularized/recellularized lungs: (1) provide appropriate surface area-volume ratios, (2) conduct blood to gas exchange interfaces while avoiding thrombus, inappropriate shear force, (3) operate using room air without a pump, (4) work with the right ventricular output without need for external blood pump (47).

### Validation and evaluation of performance

Equally as important as the design of new methods and devices is the need to validate their performance. Determination of the mechanical properties of bioengineered scaffolds and the regions of the lung they are intended to mimic using atomic force microscopy (AFM) (29, 35, 48) and microstretching techniques (49) will continue to be critical to moving forward (20). Indeed, comparative measurement of the mechanical properties of diseased tissues compared to controls is becoming increasingly important and informative. AFM is a technique that can be used to assess mechanical properties of microscale regions of lung tissue. A small tip mounted on a cantilever spring and connected to a piezoelectric positioner, laser and position-sensitive detector is applied over a surface, akin to a phonograph needle moving over a vinyl record. The vertical motion of the tip can be measured as force and magnitude as the tip is moved over the surface of the lung. The resulting force–motion data at each location, combined with the extent of the tissue deformation at the probe tip can be used to calculate the elastic modulus (from the slope of stress-strain curves). AFM data derived from bleomycin-induced pulmonary fibrosis in decellularized mouse lungs indicated a twofold increase in stiffness compared to controls but with widespread inhomogeneity (48). Although AFM is powerful for measuring material stiffness of lung tissue, it requires unique expertise, is not suitable for whole lung analyses, and is not a high-throughput technique. Estimations of mechanical properties of lung such as stiffness can be inferred by techniques that combine stretching lung tissue with microscopic examination of cells and matrix components (49). By examination of how matrix components such as collagen and elastin fibrils orient during stretch, the mechanical properties of lung tissue can be estimated. The aspect ratios of cells in the lung region being stretched can also be informative of tissue mechanical properties (50). Regardless of the techniques used to measure or estimate the mechanical properties of intact and decellularized normal and diseased lungs, further refinement of both design and data validation will be increasingly informed by mathematical modeling (51–53).

Collectively, the evaluation of the engineered tissue will require rigorous assessment of the *function* of cells and the scaffold as a system (13). The reader is directed to two excellent reviews that summarize our current knowledge of tissue-engineered models of normal and diseased lung (13, 54). In addition to function, the safety (tolerance, immunogenicity, etc.) of implanted bioengineered lungs requires careful evaluation.

## Conclusion: Challenges and Unmet Needs

The need to better treat end-stage of both adult and pediatric lung disease using newer methods and twenty-first-century technology cannot be overstated. In the United States, ~30 million people live with end-stage lung disease (55), for which lung transplantation often is the only effective treatment option. However, less than ~2,000 lung transplants are performed annually (55). The situation for children with end-stage lung disease, especially cystic fibrosis and idiopathic pulmonary artery hypertension, is even more complex (5). Recent efforts to transplant bioengineered lungs into animal models have met with tantalizing success (55, 56), but currently are not yet translatable to the clinic and no studies have yet focused on a pediatric setting. In this mini-review, we have focused on the lung, but regardless of the organ, the overarching goals for bioengineering are essentially the same and have been reviewed (22, 25). The obvious challenges to successful optimization of lung physiology such as gas exchange, nervous system innervation, ventilation-perfusion matching, and air conduction from trachea to alveolar spaces represent a huge dynamic range of physiologic and morphologic complexity. The lungs in a developing child represent additional challenges. As new technologies and approaches emerge, the bottlenecks of reproducibility and high throughput will need to be overcome. Further barriers to progress related to implantation of recellularized scaffold (57) and the potential for untoward immunogenicity (58, 59) are daunting. Finally, improving the reproducibility of the methodologies to generate decellularized scaffolds, as well as reducing intra-assay and inter-assay variability is important and worthy goals. Over the past 20 years, The National Institutes of Health, the National Science Foundation (NSF), and private foundations have dramatically increased funding for tissue engineering (search of NSF website, April 2015; http://www.nsf.gov/pubs/2004/nsf0450/inst_supp.htm). In parallel, there has been significant growth (~threefold from 2008 to 2011) in sales generated by tissue engineering and stem cell industries, estimated in 2011 to be $3.5 billion annually (60). Such dynamic economic forces will undoubtedly help the field overcome many of the daunting obstacles and deliver on past speculations made as far back as 1998: “In the next 10 years, a veritable body shop of spare parts will wend its way from labs to patients.” (61). Indeed, bioengineering the lung holds the promise to profoundly change the lives of children with intractable lung disease, from basic science, to increased efficacy of drug screens and their targets, to successful implantation of replacement lung tissue.

## Conflict of Interest Statement

The authors declare that the research was conducted in the absence of any commercial or financial relationships that could be construed as a potential conflict of interest.

## Abbreviations

ECM, extracellular matrix.
